# Verbal autopsy in health policy and systems: a literature review

**DOI:** 10.1136/bmjgh-2017-000639

**Published:** 2018-05-03

**Authors:** Lisa-Marie Thomas, Lucia D’Ambruoso, Dina Balabanova

**Affiliations:** 1 Centre for Global Development and Institute of Applied Health Sciences, University of Aberdeen, Aberdeen, UK; 2 Umeå Centre for Global Health Research, Umeå University, Umeå, Sweden; 3 MRC/Wits Rural Public Health and Health Transitions Research Unit (Agincourt), School of Public Health, Faculty of Health Sciences, University of the Witwatersrand, Johannesburg, South Africa; 4 Department of Global Health and Development, London School of Hygiene & Tropical Medicine (LSHTM), London, UK

**Keywords:** verbal autopsy, cause of death, health policy, systems research, population surveillance

## Abstract

**Introduction:**

Estimates suggest that one in two deaths go unrecorded globally every year in terms of medical causes, with the majority occurring in low and middle-income countries (LMICs). This can be related to low investment in civil registration and vital statistics (CRVS) systems. Verbal autopsy (VA) is a method that enables identification of cause of death where no other routine systems are in place and where many people die at home. Considering the utility of VA as a pragmatic, interim solution to the lack of functional CRVS, this review aimed to examine the use of VA to inform health policy and systems improvements.

**Methods:**

A literature review was conducted including papers published between 2010 and 2017 according to a systematic search strategy. Inclusion of papers and data extraction were assessed by three reviewers. Thereafter, thematic analysis and narrative synthesis were conducted in which evidence was critically examined and key themes were identified.

**Results:**

Twenty-six papers applying VA to inform health policy and systems developments were selected, including studies in 15 LMICs in Africa, Asia, the Middle East and South America. The majority of studies applied VA in surveillance sites or programmes actively engaging with decision makers and governments in different ways and to different degrees. In the papers reviewed, the value of continuous collection of cause of death data, supplemented by social and community-based investigations and underpinned by electronic data innovations, to establish a robust and reliable evidence base for health policies and programmes was clearly recognised.

**Conclusion:**

VA has considerable potential to inform policy, planning and measurement of progress towards goals and targets. Working collaboratively at sub-national, national and international levels facilitates data collection, aggregation and dissemination linked to routine information systems. When used in partnerships between researchers and authorities, VA can help to close critical information gaps and guide policy development, implementation, evaluation and investment in health systems.

**Key questions**

**What is already known?**

- Verbal autopsy (VA) is an established health surveillance method that provides information on levels and causes of death in populations where medical death certification is weak or absent.
- From 1998 to 2010, methodological and technical developments and high level advocacy have transitioned VA from use in research environments towards pragmatic applications on a wider scale, including in routine registration.
- An extensive literature exists on the validity of VA and applications in research settings. Information on its use in health policy and systems, ie on working with health authorities to provide evidence for action, is less common by comparison.

**What are the new findings?**

- Since 2010, applications of VA in health policy and systems were identified in 15 low and middle-income countries. There were various forms and extents of engagement with decision makers and governments.
- Advancements in electronic data collection, automated data interpretation and electronic databases have supported collaboration in terms of data sharing, and monitoring and evaluation of disease burdens.
- When combined with complementary methods, such as, social autopsy, the explanatory potential of VA is extended to identify contributory factors in conjunction with information on levels and causes of deaths in populations.
- Active engagement with decision makers and communities was seen to help establish a relevant evidence-base that directly informs the means for action.

**Key questions**

**What do the new findings imply?**

- VA has significant potential in health policy and systems to deliver robust and reliable evidence, help close gaps in statistics, and guide implementation, evaluation and investment.
- In conjunction with complementary data collection efforts, VA can contribute to more holistic views of health systems performance combining evidence on burden of disease with social determinants and local knowledge.
- The active collaboration of various stakeholders: governments, health authorities, communities and research groups, can foster engagement in, and coverage of, data collection and enhance the validity and utility of the process.

## Introduction

It is vitally important that data on cause of death are available, reliable, timely, and collected and aggregated at low cost for a robust evidence base to inform strategic health policy and evaluation.^1^ Collecting data on births and deaths including cause of death in high-income countries relies on well-established civil registration and vital statistics (CRVS) systems. However, in many low and middle-income countries (LMICs), there is a pervasive lack of death registration inclusive of notification of medical cause of death due to low investment over decades.^1 2^ This situation highlights an important gap, as one in two deaths go unreported globally, the majority occurring in LMICs.^1 3^

The importance of recording vital events through improved CRVS systems is well recognised. *The Lancet* series ‘*Who counts*?’ in 2007^4^ and ‘*Counting births and deaths’* in 2015^5^ emphasised the significance of robust data on vital events.^6^ There is also increasing recognition among the global health community that progress towards universal health coverage (UHC) depends on effective monitoring of equitable coverage and utilisation.^7 8^ To achieve goals such as UHC and the Sustainable Development Goals (SDGs), countries need functional CRVS systems, and the collection of data on deaths of people excluded from access to health systems is an important step for addressing health inequalities and saving lives.^9^

Verbal autopsy (VA) is a method that can be used to collect and analyse data on cause of death. VA is defined by the WHO as *‘a method used to ascertain the cause of a death based on an interview with next of kin or other caregivers’* that can be applied for deaths without certification of medical causes.^2^ Its main objective is to deliver a simple identification of cause of death at community or population level in countries where no other functional registration system is in place and/or where many people die at home without contact with the health system.^2^ Over the past 25 years, VA has become a primary source of information about cause of death in several LMICs.^2^

The development of VA can be traced back to the 1950s and 1960s, where researchers in Asia and Africa used physician interviews with relatives and carers of deceased persons to assess cause of death and generate cause of death statistics.^10^ Workers from the Narangwal project in India named this technique VA.^11^ In the 1970s, the method gained attention when WHO encouraged the use of ‘lay reporting’ by people with no medical training.^12^ Subsequently, Reproductive Age Mortality Studies, Matlab (Bangladesh) and Niakhar (Senegal) developed VA questionnaires to ascertain possible cause of death diagnosis in the late 1970s and early 1980s, which are still used in research settings and national/regional surveys.^2 13^

Concerns about the validity of instruments and comparability of data arose in the early 1990s and led to the convening of expert committees to develop standardised VA tools for childhood and maternal deaths,^14 15^ leading to the development of VA standards for maternal deaths in 1994.^16^ In 2007, the WHO published three standard VA questionnaires, ‘death of a child aged under four weeks’, ‘death of a child aged four weeks to 14 years’ and ‘death of a person aged 15 years and above’.^2^ These tools sought to permit certification and coding commensurate with International Statistical Classification of Diseases and Related Health Problems, 10th Revision (ICD-10) and ascertain all causes of death with reasonable accuracy drawing on well-administered VA interviews.^2^ The standards were updated in 2012, 2014 and 2016 to improve cause-specific mortality data and to ensure consistency and comparability between countries.^10^ Appendix 1 contains a description of the development of the WHO VA standards from 2012-2016 (online supplementary appendix 1).

10.1136/bmjgh-2017-000639.supp2 Supplementary file 2

In its present form, VA typically consists of two main stages. First, information is collected via structured interviews with family members and caregivers of the deceased on their signs, symptoms, medical history and circumstances at and around the time of death.^2^ Second, interview data are interpreted by physicians (Physician-Certified VA, [PCVA]^1 17^) or using automated methods (‘Computerised Coding of VA’ [CCVA]) for example, InterVA and SmartVA, using algorithms and probability theory,^1 18^ to obtain probable cause(s) of death.

The method is applied in a range of study designs and research settings: clinical trials and large-scale epidemiological studies, health and demographic surveillance systems (HDSS), national sample surveillance systems, and household surveys.^1 2^ The resulting data can help to determine gaps in vital statistics and help to establish population-level disease burden estimates.^19^ Furthermore, it can assist in monitoring and evaluation of health policy, planning and instituting programmes.

Due to the lack of effective national CRVS systems in many LMICs, statistics on disease burdens are often calculated by researchers using prediction models and estimation procedures.^20^ Alternative approaches to provide reliable sources of vital data are HDSS or Sample Vital Registration with verbal autopsy (SAVVY) systems. While surveys collect information from a representative population sample,^21^ HDSS and SAVVY systems aim to regularly monitor *‘demographic and health characteristics of a population living in a well-defined geographical area’* and collect prospective, longitudinal data.^20^

VA has been used to investigate population health in selected contexts by examining sequences of events in detail, particularly rare events, for example, maternal deaths.^21^ HDSSs are commonly situated in deprived rural, semiurban or urban areas where generalisability is conditional on a geographically, well-identified context.^21^ VA is frequently used in HDSS contexts to determine levels and causes of deaths for surveillance populations. Rather than precise descriptions of events at a given time, HDSS focus on the relationships between events at the community, household and individual levels over time.^21^ The information generated can help to evaluate the effects of, and give evidentiary support to, the scaling up of interventions.^20^ Furthermore, HDSS data collected and analysed with standardised tools, compared and cross-validated with national and international data, has shown extensive conformity, for example, recently with data from the International Network for the Demographic Evaluation of Populations and their Health (INDEPTH) and Global Burden of Disease estimates, which supports generalisability beyond specific surveillance sites.^21^

VA is a well established surveillance method used in over 45 LMICs for over 25 years mainly in research settings, and/or as part of large household surveys to calculate disease burdens in populations.^9^ Developments in VA tools and analysis, combined with wider recognition of the dearth of mortality data globally, have led to VA being recognised as a valuable, interim approach for use outside research environments, and towards use in CRVS systems.^22^ On this basis, this review is concerned with how VA has been used to inform health systems, policy and management in LMICs.

Major collaborative networks also support the use of VA, eg initiatives such as ‘Bloomberg Data for Health’, a network of local and national authorities in 20 LMICs, seek to strengthen the collection and use of critical public health information using the method. One of the key objectives of the initiative is to provide resources to understand broad systems issues, as well as promote the integration of VA data into CRVS systems, enhancing the use of data for maximum impact in policymaking and priority setting.^23 24^

Furthermore, the Million Death Study (MDS) in India (1998–2014), one of the largest studies of premature mortality in the world, involves the use of VA on a large scale, to influence policy and decision making. The MDS worked in collaboration with the Registrar General of India, which since 2002 has integrated an enhanced form of VA into its large-scale, nationally representative Sample Registration System.^25^ Monitoring births and deaths that occur outside healthcare facilities with VA in approximately one million randomly selected households has provided invaluable information to governments, research agencies and media, leading to action against preventable deaths.^26^

The wider use of VA is often hampered by concerns about validity. While there have been several studies investigating this issue, their findings need careful consideration. Medical autopsies and hospital records in LMICs are often of poor quality.^27^ However, these are frequently used as a ‘gold standard’ in testing the validity of VA. Validation studies are therefore a comparison of two imperfect cause of death assignments.^27^ To this end, the Population Health Metrics Consortium (PHMRC) conducted research in four countries documenting deaths in high-level hospitals and followed these up with VA, developing a data set labelled as ‘gold standard’ VA data.^28^ However, the authors acknowledged that even though the VA data might have achieved internal validity, the external validity was likely to have been limited.^28^ While VA may be seen as an imperfect tool for ascertaining cause of death, it is often the only alternative in the absence of medical certification.^22^

A further stream of methodological development aims to combine VA with social autopsy (SA). SA is a method aiming to *‘collect the data needed to connect the fatal illness or the act of diagnosing or recognizing that illness to a set of socio-demographic, economic, cultural conditions or factors’* representing a social diagnosis of the death.^29^ Relative to VA, SA is a novel approach, which has not been widely practised and still lacks standardisation for data collection and analysis to the extent seen with VA.^30^ Current work by the Child Health Epidemiology Reference Group (CHERG) and INDEPTH seeks to arrive at standardised SA tools.^31^ Methodologists posit that VA combined with SA provides enhanced data that can generate more holistic information on causes and determinants of deaths and situate cause of death in a richer account of context.^32^ In addition, ‘VASA’ (verbal autopsy and social autopsy) studies have been conducted in Bangladesh (2007–2011), Malawi (2011–2012), Niger (2012–2013) and Nigeria (2013), using retrospective surveys for data collection.^32^ VASA data have also been incorporated into updates of modelled data as part of global and regional mortality estimates, supplemented national mortality estimates, and informed policy and programme development.^32^

### Aims and objectives

Considering the methodological developments and global recognition of the broader use of VA both for research and policy development, the contribution of this paper is twofold: first, it aims to examine how VA is used to inform the operation of health systems (ie, beyond surveillance or condition-specific purposes); and second, to identify and review how VA is used in an ‘embedded’ manner in health policy and systems development. That is, with and for health authorities, aligning to and reflecting the realities of implementation^33^ and informing action. It updates a previous review by Fottrell and Byass, charting the methodological transition of VA from research environment to routine application in CRVS systems^13^ with a focus on the use of VA to inform health system and policy research and development.

## Methods

The review was conducted using a systematic search strategy. Information was gathered on implementation features, the use and purpose of VA to strengthen health policy and systems, and strengths and limitations of the approaches used.

### Search strategy

**Figure 1:**
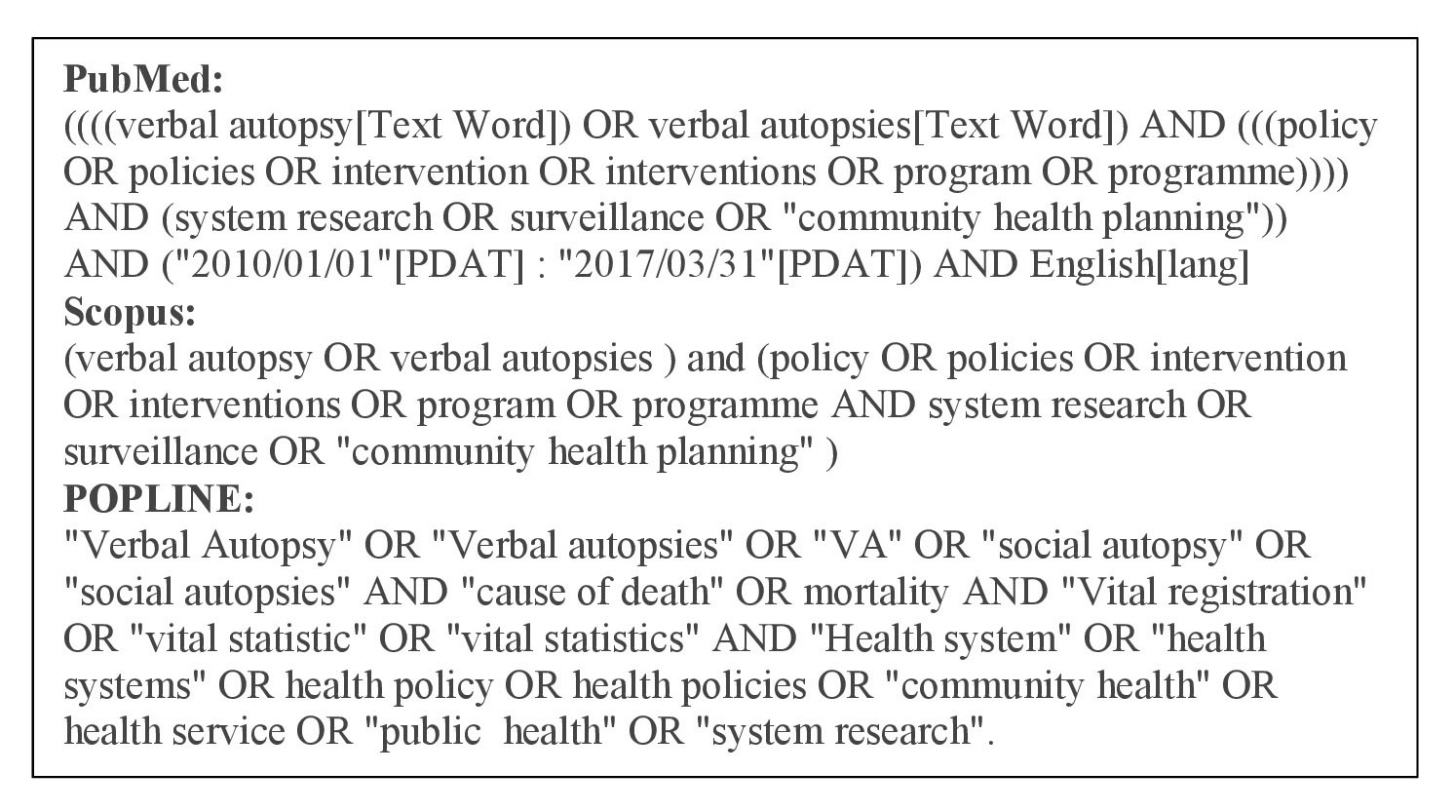
Search strategy used for PubMed, POPLINE and Scopus.

We examined online databases, including PubMed, POPLINE, Web of Science and Scopus using combinations of keywords and phrases (figure 1). Further sources and especially grey literature were identified through manual searches of references quoted in original publications. Additional hand searches and web searches were conducted on organisational portals, for example, Centres for Disease Control and Prevention, INDEPTH, CHERG and PHMRC, to identify further papers. The time frame 2010–2017 was defined as a key paper on VA considering its methodological transition was published in 2010.^13^

### Inclusion and exclusion criteria

Inclusion criteria were as follows: (1) papers published after 2010 in a journal or reports accessible through web search; (2) papers that described engagement in health systems to employ VA findings and (3) papers written in English. Studies that did not describe a contribution to health systems and policy strengthening and/or health policy and systems research by identifying needs or changes in policies and practice (eg, reforms to secure healthier communities)^34^ were excluded.

### Data extraction and analysis

**Figure 2:**
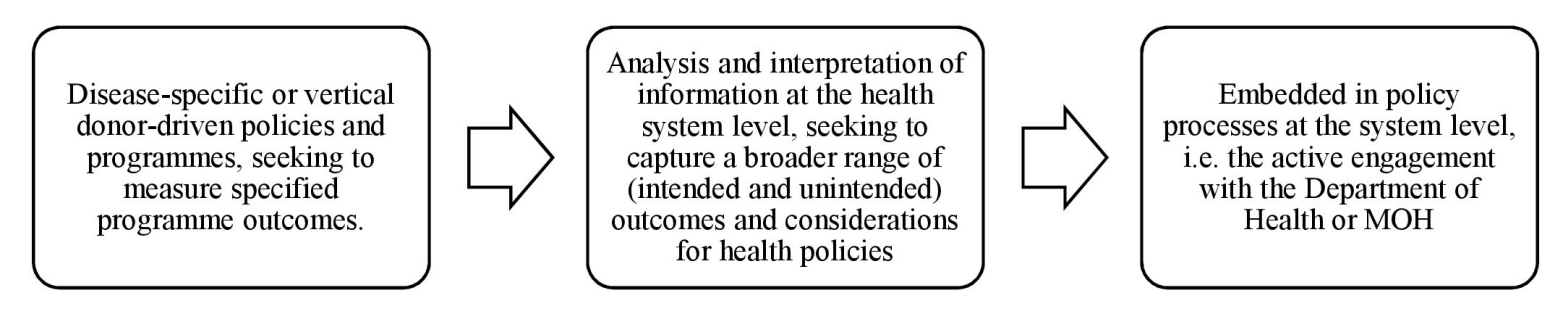
Framework: continuum of the use of verbal autopsy in health policy and systems.

A form was developed to extract data from retrieved articles on (1) study setting; (2) stated objective(s); (3) VA/SA method used; (4) remarks/limitations on the use of VA in health policy and system research; (5) applicability for health systems and policy strengthening and/or research; and (6) key findings. Articles were selected and reviewed, and data were extracted from the retrieved studies by one reviewer (LMT). Subsequently, data extraction was performed independently by two reviewers (LD and DB). Thereafter, data extractions were collated from all reviewers, and divergences were identified and resolved via discussion. Thematic analysis and narrative synthesis were subsequently conducted to analyse the data extracted. Themes and subthemes that emerged from the literature were identified, compared, discussed and associated with each other by the reviewers to ensure plausibility, validity and consistency. In accordance to the aims and objectives of the review, a framework was developed to demonstrate the extent of use of VA/SA in health policy and systems. The framework delineated a range of uses comprising: (1) disease-specific or vertical donor-driven policies and programmes, seeking to measure specified programme outcomes; (2) analysis and interpretation of information at the health system level, seeking to capture a broader range of (intended and unintended) outcomes and considerations for health policies; and (3) ‘embedded in policy’ processes at the system level, that is, active engagement with the Department of Health or Ministry of Health (MOH) (figure 2).

The methodological quality of included studies was assessed by the reviewers using the relevant Critical Appraisal Skills Programme (CASP) checklists^35^ for qualitative studies and an adapted version of the Effective Public Health Practice Project (EPHPP)^36^ assessment tool for quantitative studies. The tool usually involves rating articles (strong, moderate, weak) on six components: selection bias, study design, confounders, blinding, data collection methods, withdrawals and dropouts. In the modified version developed, the blinding section was disregarded as studies containing VA do not usually involve blinding, and so inclusion of this component would have had a misleading adverse impact on the quality assessment. The quality appraisal process sought to identify common strengths and weaknesses, rather than to exclude studies. All studies were analysed and used in the analysis with strengths and weaknesses taken into account.

## Results

### Search results

**Figure 3:**
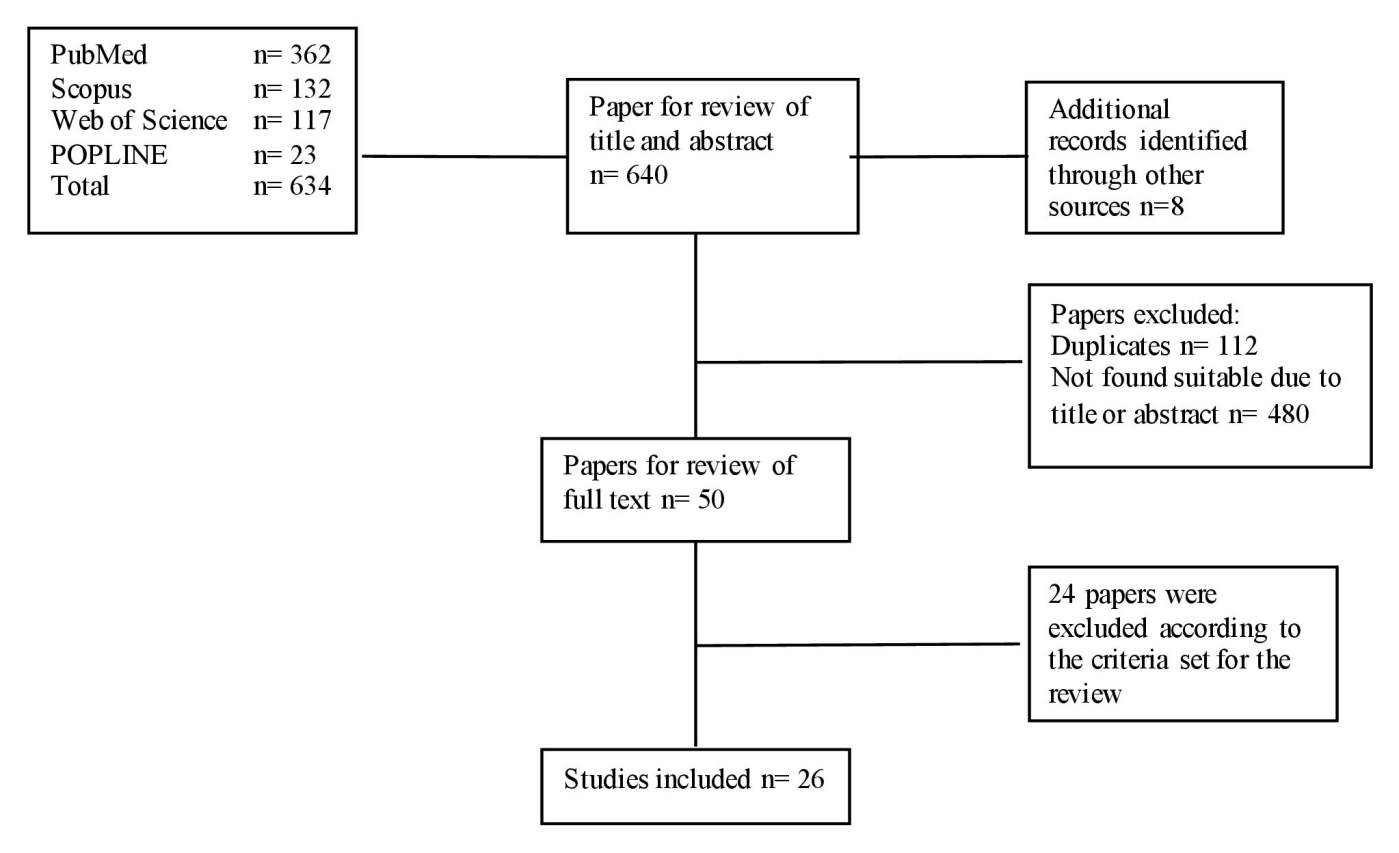
Literature search and review process.

The search identified 634 articles. 362 publications were identified through PubMed, while 132, 117 and 23 publications were identified through Scopus, Web of Science and POPLINE respectively. An additional eight papers were identified through hand searches. After title and abstract review, 26 publications were considered eligible for the review (figure 3).

### Study characteristics

**Figure 4:**
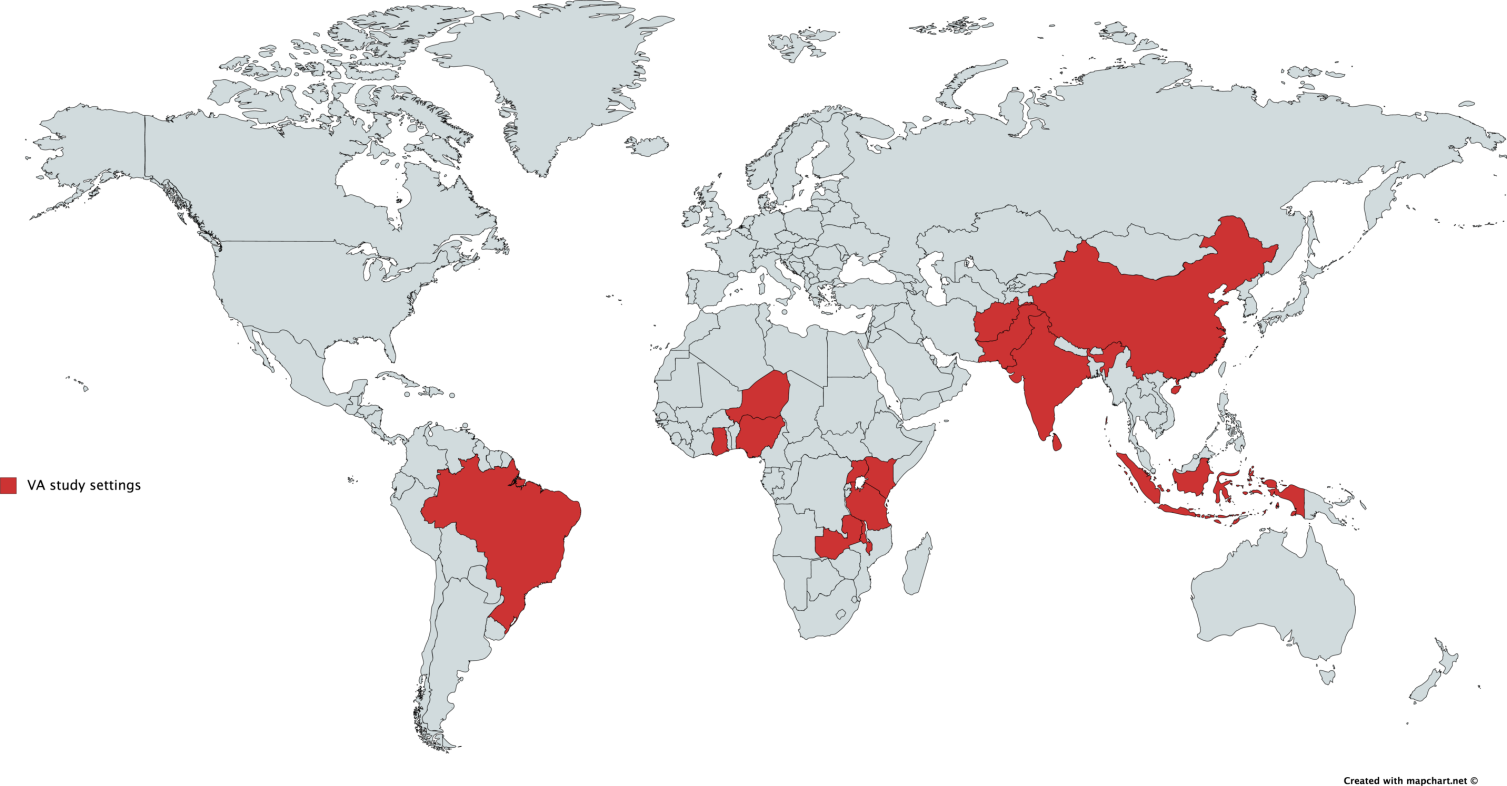
Countries in which verbal autopsy (VA) studies were conducted and reported in scientific literature 2010-2017.

From the selected papers, 14 were research papers.^22 25 37–49^ Seven were quantitative, from which the rating according to the EPHPP tool was strong for two articles, moderate for four, and weak for one.^38–40 43 45–47^ According to the CASP tool, the one qualitative study identified had a low risk of bias.^49^ Three papers were considerations of contextual factors for systems integrating VA,^22 25 44^ and two investigated feasibility issues (one of community informants and one, the cost of VA in a mortality surveillance system).^42 48^ One was a pilot study that identified weaknesses in an existing maternal death review system and introduced a community-based process to improve the recording of maternal deaths.^37^ From the remaining papers, four were HDSS profiles,^50–53^ four were correspondences/commentaries,^54–57^ two were information sheets,^41 58^ one was a summary of study results^59^ and one was a research proposal.^60^ The studies were conducted in 15 LMICs: 10 in Africa,^37 40 41 43 47 51–53 59 60^ 8 in Asia,^25 39 42 44 46 49 50 55^ 2 in the Middle East^45 48^ and 1 in South America^38^ (figure 4). In five studies, the country was not specified.^22 54 56–58^ The finding that the majority of studies took place in LMICs was not surprising as VA is predominantly used in countries where CRVS systems are incomplete or absent. Ten studies used a WHO VA tool,^38 39 43 45–48 56 58 60^ two an adapted version developed by INDEPTH,^52 53^ two used the PHMRC tool^49 59^ and one applied a study-specific tool (Vital Registration and VA tool).^40^ Eleven studies mentioned the use of a VA tool but gave no specifics on which.^22 25 37 41 42 44 50 51 54 55 57^ For data collection with VA, five stated the use of electronic devices,^40 49 52 56 60^ two used paper-based questionnaires,^42 50^ while the remaining studies did not state how data were collected. Eight mentioned the additional use of questionnaires investigating social determinants.^37 40 43 44 47 48 53 59^ Specifics of how governments were involved in the process varied considerably. In eight studies, government involvement was in more than one element of the process: five studies described involvement in planning and research programme implementation,^37–39 49 52^ and in three studies, in implementation and scale up.^45 48 59^ In a further nine studies, government involvement was described in a single element of the process: five in implementation,^25 41 43 46 47^ two in scale up,^44 60^ one in funding support,^50^ and one through a working relationship which was stated but not described,^53^ respectively (online supplementary table 1). Twelve studies were conducted within established routine health information systems,^25 38 40 41 46 49–53 59 60^ and eight introduced or established new methods of data collection on cause of death.^37 39 42–45 47 48^

10.1136/bmjgh-2017-000639.supp1 Supplementary file 1

### Implementation features

VA was used to ascertain cause of death predominantly in studies where no other form of certification was available^22 54 58 60^ or for deaths outside facilities,^25 40 45–49^ investigating mainly maternal, child and neonatal deaths.^37 41 43–45 47 48 53 59^ Other studies used VA in demographic surveillance sites in conjunction with epidemiological and demographic research,^50–53^ and for recording and investigating deaths in HDSS for the purposes of informing health policy and planning.^38 40 42 46 50–52 55 60^

Through methodological advances, and often with reference to the use of CCVA, VA was stated to have become a valued and feasible approach for use outside research settings, for wider application in health systems and policy as well as in national CRVS systems.^22 25 38 40 42 46 48 49 54–56 58 60^ This was supported by the study from Joshi *et al*,^42^ which investigated the cost of a VA-based mortality surveillance system in rural India. The study concluded that the approach was cost-effective (US$0.10 per capita per year), and that with data collection on electronic devices the cost could be further reduced.^42^ The authors demonstrated the use of VA in a mortality surveillance system as feasible, and as capable of providing valuable, timely evidence on cause of death for policymakers and health professionals.^42^

Other studies did not directly investigate costs but stated that cost savings could be achieved by conducting VA on a representative sample of all deaths or in a selection of registration administrative areas.^22^ As noted by Sankoh and Byass,^56^ the time and resource implications of establishing and maintaining CRVS systems inclusive of VA are inevitable, however the benefits that functional CRVS systems confer on people and societies are considerable. These include enabling access to public goods, facilitating development across all sectors and delivering valuable information for health policy and planning.^56^ In this sense, the cost of *not* collecting vital information is acknowledged as far higher. Government funding and commitment, as well as support and governance through an independent body, were also recommended to ensure longevity, continued evolution and provision of academic freedom to achieve this.^43 44 50^

Several studies described VA as an applicable tool that adds value across intersectoral domains and beyond public health.^38 40–42 44 50 59 60^ These included ministries of health, local governments, specialised agencies (eg, national and international organisations), national health insurance, local health authorities, health facilities, community workers as well as departments of justice and interior.^22 43 54^ Several authors recommended an interdisciplinary and intersectoral approach that combines empirical and analytical efforts with different organisations, stakeholders and the community to enable functional registration in poorly resourced health systems.^22 37 44 47 53 59^ In the paper by Bensaïd *et al*,^59^ an intersectoral collaboration is described to demonstrate the power of implementing surveillance and response interventions in settings with high levels of mortality. The collaborative effort was initiated by the government of Niger and implemented by the National Statistics Institute and a VASA working group including the MOH, the Niger country office of UNICEF and other partners in partnership with the John Hopkins School of Public Health, which provided technical assistance on behalf of WHO/UNICEF and supported by CHERG.^59^

Furthermore, communities were described as a critical foundation on which to build CRVS systems and ensure sustainability.^37 44 52 54^ So-called hybrid approaches, using a combination of reporting through community-based systems, for example, health facilities, burial authorities, key informants as well as official registration, were detailed.^22 38 58^ Several authors pointed out, however, that communities should not be ‘used’ as passive data collectors but instead adopt roles as active partners in the surveillance and response processes.^37 44 48 54^ Authors also noted that this approach has the potential to generate self-sustaining sources of information on mortality, improve trust in health systems, and counteract the potential for ’community fatigue' from repeated data collection cycles.^37 52^

In this sense, active community involvement can enhance understandings of, and ultimately transform social exclusion from, access to health systems by shifting control over the production, use and exchange of knowledge on health towards those most directly affected.^54^ One example of such an approach was a pilot study by Bayley *et al*,^37^ which investigated a community-linked maternal death review (CLMDR) to measure and prevent maternal mortality.^37^ The study demonstrated that CLMDR improved identification and review of deaths, providing opportunities for education and stimulating action in facilities. Due to the success of this approach in a sample site in Malawi, the MOH initiated a nationwide roll-out of the programme.^37^

Studies employing VA to examine facility-based deaths were also identified. VA was described to become particularly important where postmortem pathological examinations are not available, not mandatory, unreliable, or death certificates are issued for non-specific causes, which are of little value for public health decision making.^22 38 55 58^ De Savigny *et al*^22^ and Bayley *et al*^37^ note the importance of investigations of this nature when recording maternal deaths that were not attended by trained providers.

### VA in health systems and policy

Of the papers reviewed, 18 explicitly acknowledged a close intersection between the use of VA methods, and the functions of the health system to improve population health. This was achieved via: (1) active engagement of the government in the process,^39 43 46 47 49 55 59 60^ for example, high-level engagement of the Niger government and stakeholders for dissemination of VASA study results to support policy development,^59^ (2) demands from, or working relationship with, governments/health authorities,^38 45 48 50 52 53^ for example, the request from the Ministry of Public Health in Afghanistan to expand an intervention programme to reduce maternal deaths,^45^ and (3) purposeful dissemination of information to governmental, national and international agencies,^37 40 41 51 58^ for example, VA within the Millennium Global Village-Network provided feedback to local providers.^40^

In combination with questionnaires about social determinants, including as part of SA processes, VA was also used to identify social factors contributing to deaths and to determine gaps in health systems.^44 52–54^ SA has been reported as effective in supporting a more comprehensive analysis of the extent to which interventions achieve intended effects or help to explain why actions to improve people’s health are not taken by community members or health authorities.^44 47 53^

The studies demonstrated that when other routinely collected data, for example, surveys on demographic characteristics, are supplemented with VA data on a regular basis, they can provide information that can help to enhance health planning, inform policies and contribute to health system strengthening. However, to do so, data need to be shared and interpreted at all levels, from communities to systems.^38 40–42 44 46 47 52 54 59 60^

### Strengths and limitations of VA for health systems and policy

According to the WHO, the gold standard for cause of death reporting is certification by a medical practitioner trained using the ICD system.^61^ However, in LMICs there is often insufficient supply of appropriate medical equipment, trained forensic experts and pathologists. Cultural norms and traditions can also interfere with timing and performance of autopsies.^38 46^ As de Savigny *et al*^22^ state, the absence of locally relevant gold standard data presents challenges in conducting locally applicable validation studies to determine the external validity of VA results. As stated above, VA has become increasingly standardised in recent years and more frequently applied globally, allowing for comparison with other standardised methods, for example, hospital records.^56 58^ The literature also acknowledged, however, the value of adaptations to context, and that standardisation may inadvertently compromise accuracy, completeness and validity, limiting relevance to local policy and planning.^22 49 54 58^

Use of mobile devices and electronic databases, ‘mHealth’, was a relatively new development affecting how VA is used. Electronic platforms were frequently described as able to enhance data sharing and link data of a range of types and sources: demographic, epidemiological, mortality, morbidity, clinical, laboratory, household, environmental, health system for a more holistic view.^57^ National electronic records systems, ‘SmartCare’ in Zambia,^62^ for example, have been reported to create new opportunities to enhance the timeliness of data collection and analysis, among others with VASA, enabling real-time data capture.^22 40 47 52 53 60^ These advances were reported to assist in the detection of rapid changes in health systems and disease burdens, as well as track key indicators related to local, regional and global goals and associated targets,^48 59^ aiding in efficient decision making and delivery of care.^25 40^

Encouraging the use of VA in countries lacking adequate or representative data via the expansion of HDSS, and with VA complementing hospital data were also seen as promising approaches.^22 38 41^ De Savigny *et al*^22^ recommend that data gathered with VA in the community should be analysed with physician-certified hospital deaths. To realise potential, it was further recognised that expansion of infrastructure and collaboration between research and service organisation and delivery are necessary.^54^ In South Africa, for example, the Department of Science and Technology has recently launched an initiative to consolidate three existing HDSS sites and expand the platform with three further urban and rural nodes, to cover over 1% of the national population.^63^

## Discussion and conclusion

This review sought to examine how VA is used in a manner consistent with the emerging health policy and system research paradigm.^33^ The review fills an important gap in the literature on how VA is (and can be) used to support the routine operation of health systems, not just in terms of surveillance but in policy and programme development. Further, it explores its use in an ‘embedded’ manner in health policy and systems research, working with planners and implementers.

A number of limitations of this review need to be acknowledged. Findings may have been influenced by publication and reporting bias. The search may have missed studies in which VA was used in health policy and systems due to the lack of uniform reporting or indexing methods. Many key studies are also reported in the grey literature, with uneven quality and variable availability. On this basis, grey literature was included to increase the breadth, relevance and utility of the review.

The studies identified were conducted in 15 LMICs in Africa, Asia and South America using mostly standardised VA tools to investigate generally maternal and child mortality. Of the studies selected, eight used VA ‘embedded’ in projects investigating the effectiveness of single or multiple interventions implemented in the study area. Four used VA in established HDSS sites to ascertain cause of death in the population, while the remaining studies looked at the feasibility of VA in national health and vital registration systems.

The studies either disseminated data to health authorities and government representatives or stated the direct involvement of a government representative in the process (ie, in planning, implementation and/or scale-up). A notable shift in the time period covered by this review was the application and use of VA beyond research settings. The method has been developed to enhance appropriateness and feasibility of implementation in vital registration where information gathered is actively and routinely used by health authorities. The review revealed a continued transition of the VA method beyond research environments into health policy and planning, contributing to achievements in, and demonstrating of progress towards UHC and other targets. With methodological advancements and technical developments, it is regarded as a valuable and feasible approach to supplement and strengthen national sample and CRVS systems in LMICs.^22 38 49 58^ The increasing use of electronic means to collect, compute and share real-time data, for example, using tablets, CCVA, e-health and mHealth recording systems, is also contributing to development and use on a wider scale and routine basis.^22 49 62 64^

An intersectoral focus and the involvement of communities were reported as further key developments to the method, which were seen to facilitate sustainability. Here an important tension was identified whereby despite efforts to standardise and generate comparable data across settings, it was noted that VA is not a one-size-fits-all approach,^20^ and adaptations to local conditions were described as critical for maintaining relevance to national and local health contexts.^10 13^ Standardised and validated VA were also described as important for evidence-based resource allocation when used by regional, national, cross-national and international actors.^65^ Moreover, when VA is used in conjunction with other data collection methods, for example, SA on demographic and health system characteristics, a more holistic view of health systems performance was achieved.

Improving CRVS systems in LMICs was described to involve national policymakers and governments, and to require commitments from the global health community to help fund and build these systems. This was seen to require an interdisciplinary and intersectoral effort involving a variety of organisations and stakeholders such as MOHs, ministries of local government, national and international agencies, as well as health facilities and researchers. There was also evidence of merit in the involvement of communities and families living in registration sites.^37 44 52 54^ Several partnerships models were seen to help extend CRVS systems in settings lacking resources and capacity, fostering improved health systems performance and, ultimately, reductions in avoidable mortality.^22 38 58^

There is a growing demand for evidence-based and data-driven interventions in LMICs. With new technologies facilitating data collection and analysis, and in combination with supplementary data collection methods, VA is used in a versatile manner beyond research settings to inform policy, programming and planning. Where governments are involved in the planning, implementation and/or scale-up of VA activities, information tended to be used more directly in planning and policy processes. Increasing coverage and use of VA on a routine and continuous basis may offer information gains on many fronts: to inform local health institutions and policy development, and in planning and research at national and global levels. Data that are developed collaboratively and communicated effectively, recognising strengths and limitations, were further promising developments. The literature reviewed suggests that VA has potential to help close the gaps in the availability and use of intelligence, and guide policy implementation, evaluation and investment in health systems.
