# Supplementary material for: Verbal autopsy in health policy and systems: a literature review
Source: BMJ Glob Health. 2018 May 3;3(2):e000639. doi: 10.1136/bmjgh-2017-000639 (PMC5935163; doi:10.1136/bmjgh-2017-000639)
Supplement: Supplementary file 2 [file bmjgh-2017-000639supp002.pdf]

Appendix I: Development of the WHO VA standards from 2012-2016

Development of the WHO VA standards (according to the WHO guidelines) from 2012	
2012	Changes mentioned by WHO:<br>Shortened VA questionnaire<br>Questions are reduced and reformulated to 'yes' and 'no' answers<br>Contains 192 cause of death related indicators, subdivided into 4 sections and 118 subgroups<br>Skip patterns within sections and subgroups are driven by age, sex, maternal and perinatal information<br>Designed for software use<br>General Information:<br>Personal information (on the deceased; vital registration)<br>Information on the respondent;<br>COD (medical history; general signs and symptoms; Signs and symptoms associated with pregnancy; Neonatal and child history, signs and symptoms; History of injuries and accidents; Risk factors; Health service utilization)<br>Background and context<br>Recommended optional open narrative text<br>Additional Questions:<br>Questionnaire 2: Pregnancy related questions for 12 to 14-year-old female children<br>Questionnaire 3: Includes section for all female deaths<br>The 2012 WHO VA instrument gives also suggestions on how to use it in routine vital registration.<br>Link to WHO 2012 instrument:<br>http://www.who.int/healthinfo/statistics/WHO_VA_2012_RC1_Instrument.pdf?ua=1<br>[17,18]
2014	Changes mentioned by WHO:<br>Simplified instrument<br>Contains 265 COD related indicators of which only subsets are used in the different age groups – 4 sections, 118 subgroups<br>General Information:<br>Personal information (on the deceased; vital registration and certification)<br>Information on the respondent;<br>COD (medical history associated with final illness; general signs and symptoms associated with final illness; Signs and symptoms associated with pregnancy and women; Neonatal and child history, signs and symptoms; History of injuries and accidents; Risk factors; Health service utilization)<br>Background and context<br>Recommended optional open narrative text<br>Additional Questions:<br>Questionnaire 1 and 3: Pregnancy related question for 12-14-year-old female children<br>Link to WHO 2014 instrument:<br>http://www.who.int/healthinfo/statistics/verbalautopsystandards/en/<br>[11,66]

2016	Changes mentioned by WHO:<br>New and edited questions are integrated in the tool to better facilitate the use of available analytical software for assigning COD (SmartVA, InterVA, InSiliconVA)<br>Editing of a skip pattern<br>Free text fields are included to allow information<br>Review of the comparability with SmartVA and InterVA<br>General Information:<br>Information about the prevalence of malaria and HIV<br>Information about the respondent, consent and time of interview<br>Information about the deceased (Socio-demographic can civil registration information)<br>History and details of injury/ accidents<br>Medial history associated with the final illness (Duration of final illness, history of diseases likely to be associated with or the COD, general signs and symptoms relevant for maternal deaths, signs and symptoms relevant for neonatal and child deaths, health service and contextual factors, information from death certificate)<br>Narrative description of final illness (text field)<br>Check list of key indicators from the narrative description<br>Link to WHO 2016 instrument:<br>http://www.who.int/healthinfo/statistics/verbalautopsystandards/en/<br>[2,9]
