# Supplementary material for: Verbal autopsy in health policy and systems: a literature review
Source: BMJ Glob Health. 2018 May 3;3(2):e000639. doi: 10.1136/bmjgh-2017-000639 (PMC5935163; doi:10.1136/bmjgh-2017-000639)
Supplement: Supplementary file 1 [file bmjgh-2017-000639supp001.pdf]

Table 1. Study settings and tools

Reference	Author	Study Setting	Population and Volume of data	Data collection and government involvement	Why Verbal Autopsy (VA)	Tools used	VA analysis	Authors' opinion of the methods used	Health system aspects
[24]	Aleksandrowicz et al., 2014	Part of the Million Death Study in India.	All deaths. Volume of data is based on data of the Million Death Study.	Secondary data. Government was involved in the conduction of the study.	To investigate the underlying Cause of Death (COD). Routine, reliable, representative, resampled household investigation of mortality with medical evaluation.	Performance criteria were set which included:<br>(i) A low ill-defined proportion of death before old age;<br>(ii) Reproducibility (including consistency of COD distributions with independent resampling;<br>(iii) Differences in COD distribution of hospital, home, urban or rural deaths;<br>(iv) Age-, sex- and time specific plausibility of specific diseases;<br>(v) Stability and repeatability of dual physician coding; and<br>(vi) The ability of the mortality classification system to capture a wide range of conditions.<br>The World Health Organization (WHO)-VA standard was used and data was collected electronically.	Physician-certified Verbal Autopsy (PCVA).	Simple VA systems that obtain a random sample of deaths will offer an attractive option over the next few decades as despite the misclassification of VA-based COD systems, they better than the current data on COD.	The use of VA in the MDS demonstrates that national surveys of CODs using VA are an order of magnitude better than the limited COD data previously available. Key implications for low- and middle-income countries (LMICs) were medical certification of deaths is lacking is to conduct random sample surveys which include VA to investigate deaths outside healthcare facilities.

[21]	De Savigny et al., 2017	No setting specified.	No specified population.	No data collection or government involvement.	To integrate community-VA into Civil Registration and Vital Statistics (CRVS) systems.	About general use of VA.	Not specified.	VA is an imperfect tool for ascertaining COD but it is the only alternative in the absence of medical certification. Using electronic questionnaires for data collection and computer algorithms to analyse responses and estimate probable COD have increased the potential for VA to be routinely applied in CRVS systems. However, an array of stakeholders and sub-systems have to be addressed which are integrated with existing CRVS work processes and information flows. VA results have to be linked to civil registration records, information technology requirements and data quality assurance.	Integrating VA into CRVS systems can have profound system-wide effects that need to be addressed before implementation.

[37]	Bayley et al., 2015	National sample, Malawi, Mchinji District.	Maternal deaths 52 Maternal deaths, from which 45 were subject to some review, 37 were discussed in Community Linked Maternal Death Review (CLMDR) meeting, 35 in the bimonthly review meeting. 28 completed all five stages of the CLMDR process.	Primary data collection. Study in partnership with the Ministry of Health (MOH) Malawi which helped design, pilot and roll-out the CLMDR system.	To obtain data (from VA and other methods used) to facilitate discussion and communication between participants in the meetings held in combination with the project. Furthermore, to inform actions.	Five stages:<br>(i) Community based VA (paper-based);<br>(ii) Community review meetings;<br>(iii) Facility review meetings;<br>(iv) Public meeting/community feedback meeting;<br>(v) Bimonthly progress meeting.<br><br>No statement was given on what VA standard was used.	Health workers.	Maternal death surveillance systems can help to identify maternal deaths outside the health care facility.	CLMDR can help to measure, identify and, review data on medical cause of death which are essential for health services to monitor changing patterns of maternal mortality and response to health interventions.
[38]	Franca at al., 2011	National sample. Brazil, northern region of Minas Gerais state.	All deaths recorded in 10 municipals in Brazil. 202 eligible ill-defined deaths form which 151 were investigated with VA.	Secondary data.<br>In 2007 the MOH considered the adaption of an adapted WHO-VA questionnaire for the investigation of ill-defined COD. In some parts of Brazil VA is used as a part of the Government initiative to strengthen the vital registration system.	To further investigate death certificates with ill-defined conditions.	WHO-VA standard. No statement on how data was collected.	PCVA as well as medical certificates or any other relevant documentation that might help determining the death.	VA is an important tool in the investigation of ill-defined conditions existing in vital registration systems with data quality problems.	The data collected with VA should be highlighted to regional health policymakers to improve the quality of data for improving policy planning.

[39]	Pane et al., 2013	Indonesia	All deaths of Indonesian Hajj Pilgrims.	Secondary data collection. Government initiated study and help planning and implementing it.	To identify patterns of COD for Indonesian pilgrims and comparison between routine death certificates to VA findings.	WHO-VA standard. No statement on how data was collected.	Trained staff from the Ministry of Health.	Further studies are needed to assess the utility of VA in this setting.	The correct classification of deaths is critical to target preventive interventions and provide health service. The study was initiated by the MOH in order to evaluate the effectiveness of pre-departure health screening, exclusion of the severely ill and the provision of appropriate drug therapies.
[40]	Ohemeng-Dapaag et al., 2015	National sample, Ghana.	Millennium Villages Project (MVP); adult, maternal and child deaths, Volume of data not stated.	Secondary data collection. No government involvement stated.	To identify deaths in projects.	(i) Vital registration with VA (VRVA) tool (not standardised, during study time, MVP was testing entry on Java-enabled mobile phones);<br>(ii) Birth registration form;<br>(iii) Cause of death module;<br>(iv) Social autopsy module;<br>(v) Community Morbidity & Mortality Rounds.	Algorithm.	Integration of vital registration and VA with Millennium Global Village-Network (MGV-Net) information system makes it possible for rapid assessment of effectiveness and provides important feedback to local providers.	States that the integration of VA into vital registration with the MGV-Net information system makes a rapid assessment of effectiveness possible and provides important feedback to local providers and MVP.
[41]	Konopka and Levine, 2015	Regional roll-out, Malawi.	Maternal deaths, Volume of data not stated.	Primary data collection. Government was involved in the implementation of the project.	To record maternal deaths outside health facility.	Maternal Death Surveillance Response (MDSR) guidelines including VA. No statement on how data was collected.	Not specified.	No opinion stated.	MDSR systems was successfully integrated with the national Integrated Disease Surveillance and Response program. The VA tools and approach developed were adopted and included in the national MDSR Guidelines.

[42]	Joshi et al., 2015	National sample, India.	5895 deaths were identified from which 5786 were investigated with VA.	Primary data collection. Government involvement not clearly stated.	To investigate the cost of implementing VA in mortality surveillance system.	Adapted questionnaire, no further specification. Data collection was paper based.	PCVA.	For the population studied it is feasible to establish and run a VA based mortality surveillance system for about USD 0.10 per capita per year.	The study concludes that is it feasible establish and run a VA based mortality surveillance system in rural India.
[43]	Orobaton et al., 2017	National sample roll-out, Nigeria.	11 maternal deaths were identified form 36,370 mothers which were reached with misoprostol. All deaths were investigated with VA.	Primary data collection. Government was involved in implementation and scale up of the project. It introduced a community-based system for distribution.	To follow-up maternal deaths.	(i) Simple patient outcome form;<br>(ii) Data collection at community level through observation and questioning;<br>(iii) Monthly review meetings;<br>(iv) WHO-VA standard. No statement on how data was collected.	PCVA.	VA findings underscore the need to continue advocacy for improved emergency transport systems and their use, and improved quality of care in facility-based deliveries for all mothers.	The study provided technical support to the Sokoto State Government to initiate and expand community-based delivery of misoprostol state-wide, at scale.
[44]	Kalter et al., 2011	National sample, India, eight states.	229 maternal deaths were investigated with VA.	Secondary data collection. Government was partially involved in up-scaling of project.	To conduct confidential inquiries of recent maternal deaths to gather information on their causes and contributors.	Conceptual Framework<br>(i) Maternal health policy objectives;<br>(ii) Contextual factors;<br>(iii) Community factors;<br>(iv) Public health systems factors;<br>(v) Governance factors.<br>No statement of what VA tool was used or on how data was collected.	Not stated.	Findings support hypothesis that contextual factors strongly affect the success of a maternal death inquiry and response process in helping to meet critical maternal health policy objectives.	The Maternal and Perinatal Death Inquiry and Response (MAPEDIR) was implemented in 18 districts across eight states in India with the aim of addressing critical policy issues affecting maternal mortality. The study examined the up-scaling of the process and how this was influenced by state-specific contextual factors that affected MAPEDIR's ability to fulfil the related policy objectives.

[45]	Haver et al., 2016	National sample, Afghanistan, 20 districts.	22 maternal deaths were identified form which 16 were investigated with VA.	Primary data collection. The Government helped with implementation and roll-out of the study.	To investigate deaths of pregnant women or women who had recently given birth, to investigate effectiveness of Misoprostol intervention.	(i) Surveys pre- and post-intervention; (ii) WHO-VA standard; (iii) Maternal death audits. No statement on how data was collected.	PCVA.	The study builds on findings of a pilot study and demonstrates the effectiveness and safety of a program for advanced distribution of misoprostol for self-administration to prevent postpartum haemorrhage. Experiences made can inform maternal health strategies.	The Ministry of Public Health (MoPH) in Afghanistan requested that the US Agency for International Development-funded Health Services Support Project expand the intervention to confirm program effectiveness, including identification of adverse events, before incorporating it into national policies. Based on the results the MoPH committed to the further expansion of the program.
[46]	Rao et al., 2010	National sample, Java, Indonesia.	All deaths. A total of 10,038 were identified. However, no clear distinction which were investigated with VA or through other sources.	Secondary data collection. The Government was involved in the implementation of the project.	To investigate deaths outside hospitals. VA based death certificates from health centres were submitted to the district health office where trained coders applied rules to select and code the underlying cause for each death.	(i) Inter-sectoral working group reviewed existing death registration procedures, and proposed a streamlined mechanism for death notification and registration at local level; (ii) Baseline population data were obtained from the local offices; (iii) Deaths in hospitals, physicians issued a death notification form for deaths in hospitals to relatives, and completed standard medical	PCVA.	Continued implementation of the Indonesian Mortality Registration System Strengthening Project (IMRSSP) over the next 5-10 years, supported by appropriate operational research activities to measure and improve data quality is necessary to develop a routine source of mortality data for health policy and monitoring.	IMRSSP was launched in 2006, towards developing a routine data source. The study reports about the methods and processes developed and implemented in the IMRSSP as well as an analysis of data collected.

						certificates of COD;<br>(iv) For deaths outside hospitals, local health centre a WHO-VA standardised tool was used.<br>No statement on how data was collected.			
[47]	Serbanescu et al., 2017	National sample, Uganda and Zambia.	Maternal deaths. Maternal Mortality Ratio based on information collected through VA: In Uganda in the baseline Reproductive age mortality studies (RAMOS) 452 were identified and 316 in the 1-year RAMOS. A total of eleven deaths could not be followed up with VA.	Primary and secondary data collection. Government was involved in implementation of the initiative.	To investigate deaths of women who died while pregnant or postpartum.	Saving Mothers, Giving Lives (SMGL) –<br>(i) Health facility assessment;<br>(ii) Pregnancy outcome monitoring in facilities;<br>(iii) Rapid ascertainment process for institutional deaths;<br>(iv) RAMOS – VA and Social Autopsy (SA) questionnaire featured in the 2013 WHO technical guidance – only conducted in Uganda).<br>No statement on how data was collected.	PCVA.	Lessons learned from SMGL can inform policymakers and program managers in other LMICs where similar approaches could be utilised to rapidly reduce maternal mortality.<br>The study helped to identify specific areas that need strong policy and programmatic interventions to improve maternal health outcomes.	The study looked at the SMGL initiative that serves as a 'proof of concept' and as the basis for scale-up in Uganda, Zambia and potentially additional countries. The results demonstrate, that a comprehensive health-system strengthening approach with improvement to access to, availability, and quality of maternity care in the SMGL districts in the first-year lead to a decline in population- and facility-based maternal mortality in both countries.

[48]	Mir et al., 2015	National sample, Pakistan.	Maternal deaths. 2001 deaths of women of reproductive age from which 1808 were investigated with VA and 1424 met the inclusion criteria of the study.	Secondary and primary data collection. The Government was involved in implementation and roll-out of the study.	To identify deaths which then were followed up with VA to estimate maternal mortality in order to investigate feasibility of key informants.	Five study phases: (i) Identifying networks; (ii) Assembling the Networks (MADE-IN); (iii) Follow-Up of Deaths (MADE-FOR) - WHO-VA standard including questions about socioeconomic characteristics, health seeking behaviour, quality of care (No statement on how data was collected); (iv) Estimating the Probability of Capturing Deaths by the Informant Networks.	Inter-VA.	Pilot study added to the available evidence that shows reliable information on deaths among women of reproductive age can be obtained at a reasonably low-cost from community-based networks.	The study shows that it is feasible and economical to use community information to identify recent deaths and when followed up with VA, obviate the need for conducting large-scale surveys.
[49]	Zhang et al., 2016	Luquan County, Hebei China.	All deaths during the study period. 268 deaths took place from which 227 were investigated with VA.	Secondary data. The Government of China introduces a Sample-based Disease Surveillance Point system which uses VA-questionnaires.	To investigate deaths in the Disease Surveillance Point system. The study looks at the feasibility of using the Populations Health Metrics Research Consortium (PHMRC) mobile phone-based shortened VA questionnaire.	(i) Conducting interviews with township health care providers and village doctors to carry out smartphone-based short-version VA; (ii) Qualitative interviews to evaluate the acceptability of the new method.	Algorithms.	In order to improve national health evaluation and program planning, it is essential that phone based shortened VA (msVA) is broadly implemented to increase the coverage and quality of COD data across rural China.	The study states that it is feasible to implement msVA. However, willingness and training of village doctors on the VA protocol are necessary to make the implementation successful. MsVA while more expensive than the existing paper-based version in Disease Surveillance Point systems offers an opportunity to obtain higher quality data for COD in rural, resource-limited areas.

[50]	Ghosh et al., 2015	National sample, West Bengal, India.	All deaths. VA was conducted on 841 deaths.	Primary data collection. Government founds the Health and Demographic Surveillance System (HDSS) site.	To record deaths in HDSS sites occurring at all ages routinely since 2012.	Demographic and Socioeconomic surveys; no specific VA tool mentioned. Data was collection was paper based.	Not specified.	No opinion stated.	Health system research involves studying access, equality and utilization of healthcare services, health insurance and health expenditure.
[51]	Beguy et al., 2015	National sample, Kenya, NUHDSS.	All deaths. Crude death rate (CDR) in 2012 was 6.2 per 1000 person-years from a total population of 63.639. VA was conducted on 97% of all deaths in 2012.	Primary data collection, no specific government involvement stated.	To record all deaths in the HDSS site.	Surveys on key demographic and health events. No specific VA tool mentioned. No statement on how data was collected.	Inter-VA.	No opinion stated.	Findings from the HDSS site and further studies have been influential and extensively used in policy and program discourse by donors and international agencies in Kenya and regionally in order to advocate for proactive policies that address the plight of millions of poor urban residents in the region.
[52]	Mrema et al., 2015	National sample, Tanzania, Rufiji HDSS.	HDSS site, all deaths. CDR was 8.0 per 1000 person-years from a total population of 103,503.	Primary data collection. Implementation of the HDSS was component of Tanzania Essential Health Intervention Project in collaboration of MOH and social Welfare and International Development Research Center (IDRC). The HDSS site was established as a sentinel site and to provide Health System observations to monitor effects of changes in health policies and services.	To record all deaths and socioeconomic status in the HDSS site.	(i) Surveys;<br>(ii) International Network for the Demographic Evaluation of Populations and their Health in Developing Countries (INDEPTH)-VA standard. Data collection on tablets and OpenHDS.	Algorithm.	No authors opinion stated.	The HDSS site collects information on health status and demographic indices (birth, death etc.) and offers the opportunity to conduct programs that focus on health system strengthening, social drivers of health, and health system research.
[53]	Gyapong et al., 2013	National sample, Ghana, Dodowa HDSS.	All deaths. Total population was 111,976 with a CDR of 6.5 per 1000 per year.	Primary data collection. HDSS has a working relationship with the local Government.	To record all deaths in the HDSS site.	(i) Surveys;<br>(ii) INDEPTH-VA standard;<br>(iii) INDEPTH-SA standard.	PCVA.	No opinion stated.	The HDSS site uses biometric data enrolment to further enhance

									health facility and population-based data linkage.
[54]	D'Ambruoso et al., 2017	No study setting.	No specified population.	No data collection or government involvement.	Not specified.	About general use of VA.	Not specified.	VA combined with SA can be seen as an opportunity to better understand social exclusion from access to health systems. VA can have a potentially important role in connecting information on the health of people to local health system stakeholders.	The employment of VA methods for health system strengthening approaches can help to inform local change and thereby achieve aggregated global impact. VA as a pragmatic approach in LMICs to assign COD levels where death registration is incomplete.
[55]	Dharmaratne et al., 2011	Sri Lanka.	All deaths, regional roll-out.	No data collection. No government involvement stated.	To complement the Death Registration System in Sri Lanka as majority of death notifications will not have a medically determined COD.	WHO-VA standard. No statement on how data was collected.	Not stated.	Limitations of VA are that only a limited number of diseases are included that encompass very broad categories, symptoms asked are limited as well as the ability of Death Registrars to identify the correct disease using VA.	Introducing VA with the support of policymakers is an important step towards improving the quality of COD data The WHO standard combined with automated methods for diagnosing COD has the potential to substantially improve quality and timeliness of critical COD information for policy and planning.
[56]	Sankoh and Byass, 2014	No setting specified.	No study population.	No data collection. No government involvement stated.	Not specified.	WHO-VA standard. Hand-held devices were seen as beneficial as they can bring greater integration within CRVS systems.	Not specified.	Although initiation of CRVS systems, including VA, is bound to be expensive and time-consuming, it is crucial to approach the CRVS systems as a virtuous cycle that not only requires resources but also	HDSS sites should be established and experience shared with policymakers and stakeholders to strengthen national CRVS capacities and make the use of VA more feasible.

								yields positive results.	
[57]	Sankoh, O., 2015	No setting specified.	No study population.	No data collection. No government involvement stated.	To collect mortality data from health facilities (with medical certificates of COD) and from the community.	No statement about what VA standard or data collection tool should be used.	Not stated.	The Comprehensive Health and Epidemiological Surveillance System (CHESS) can provide complementary data to national health management and information systems and link to local civil registration systems, enabling access to birth and death registration and certification for population. Thus, providing individuals and families with direct benefits.	The CHESS innovation will include monitoring health systems and policy initiatives as they affect communities and households.
[58]	UNECA, 2015	No setting specified.	No study population.	No data collection. No government involvement stated.	To collect COD data of deaths that occurred outside of health facilities.	WHO-VA standard. Not stated on how data should be collected.	Not specified.	No opinion stated.	Laws should make the assignment of COD that occur outside the health facility compulsory and if no registration system is available laws should allow the recording of COD through VA.
[59]	Bensaïd et al., 2016	National sample, Niger.	605 neonatal deaths and 605 infant and child deaths were investigated with VASA.	Secondary data collection. Government was involved in implantation and roll-out of the study.	To determine the biological COD and the CHERG SA questionnaire to collect data on the social determinants.	PHMRC standard; CHERG SA standard. Not stated how data was collected.	Algorithm and one physician.	VASA study allowed health authorities in Niger to better understand COD and constraints to accessing and utilising care, as well as weaknesses of the health system in ensuring optimal responses to the health problems of	Paper summarises the experience in Niger with VASA and highlights the steps taken for high-level engagement of the Niger government and stakeholders for the wide dissemination of the study results and their use to support policy development and maternal, neonatal and

								mothers and children. Continued input by technical and financial partners is still needed to stimulate the full utilisation and integration of the VASA tool into the National Health Information system and the National System for Development of Statistics for the monitoring of development and implementation of various recommendations.	child health programing in the country.
[60]	CDC, 2017	National sample, Kenya: Homa Bay (Phase 3 of the project would be a roll-out to selected sub-counties throughout Kenya).	All deaths shall be investigated in the catchment area.	Primary data collection. Government (MOH and Civil Registration Department) is planned to be involved in scale-up of the project.	To implement community VA which ought to be linked to a national CRVS system.	WHO-VA standard; Open data kit (ODK)-based data collection platform on smart phones.	Inter-VA.	No opinion stated.	VA provides information on causes of community deaths and enables mortality surveillance for public health decision making. It can help enhancing national mortality surveillance and can provide routine information on community deaths.

Abbreviations:
CDC, Centres for Disease Control and Prevention; CDR, Crude Death Rate; CHESS, Comprehensive Health and Epidemiological Surveillance System; CLMDR, Community Linked Maternal Death Review; COD, Cause of Death; CRVS, Civil Registration and Vital Statistics; HDSS, Health and Demographic Surveillance System; IDRC, International Development Research Center; IMRSSP, Indonesian Mortality Registration System Strengthening Project; INDEPTH, International Network for the Demographic Evaluation of Populations and their Health in Developing Countries; LMICs, Low- and middle-income countries; MAPEDIR, Maternal and Perinatal Death Inquiry and Response; MDSR, Maternal Death Surveillance response; MOH, Ministry of Health; MoPH, Ministry of Public Health; MsVA, phone based shortened VA; MVP, Millennium Villages Project; ODK, Open Data Kit; PCVA, Physician-certified Verbal Autopsy; PHMRC, Populations Health Metrics Research Consortium, RAMOS, Reproductive Age Mortality Studies; SA, Social Autopsy; SMGL, Saving Mothers, Giving Life; VA, Verbal Autopsy; VRVA, Vital registration with VA; WHO, World Health Organization.
